# Systemic Fairness for Sharing Health Data: Perspectives From Swiss Stakeholders

**DOI:** 10.3389/fpubh.2021.669463

**Published:** 2021-05-05

**Authors:** Lester Darryl Geneviève, Andrea Martani, Thomas Perneger, Tenzin Wangmo, Bernice Simone Elger

**Affiliations:** ^1^Institute for Biomedical Ethics, University of Basel, Basel, Switzerland; ^2^Division of Clinical Epidemiology, Geneva University Hospitals and University of Geneva, Geneva, Switzerland; ^3^University Center of Legal Medicine, University of Geneva, Geneva, Switzerland

**Keywords:** data sharing, systemic fairness, legislation, attribution mechanisms, health policy, health data

## Abstract

**Introduction:** Health research is gradually embracing a more collectivist approach, fueled by a new movement of open science, data sharing and collaborative partnerships. However, the existence of systemic contradictions hinders the sharing of health data and such collectivist endeavor. Therefore, this qualitative study explores these systemic barriers to a fair sharing of health data from the perspectives of Swiss stakeholders.

**Methods:** Purposive and snowball sampling were used to recruit 48 experts active in the Swiss healthcare domain, from the research/policy-making field and those having a high position in a health data enterprise (e.g., health register, hospital IT data infrastructure or a national health data initiative). Semi-structured interviews were then conducted, audio-recorded, verbatim transcribed with identifying information removed to guarantee the anonymity of participants. A theoretical thematic analysis was then carried out to identify themes and subthemes related to the topic of systemic fairness for sharing health data.

**Results:** Two themes related to the topic of systemic fairness for sharing health data were identified, namely (i) the hypercompetitive environment and (ii) the legal uncertainty blocking data sharing. The theme, hypercompetitive environment was further divided into two subthemes, (i) systemic contradictions to fair data sharing and the (ii) need of fair systemic attribution mechanisms.

**Discussion:** From the perspectives of Swiss stakeholders, hypercompetition in the Swiss academic system is hindering the sharing of health data for secondary research purposes, with the downside effect of influencing researchers to embrace individualism for career opportunities, thereby opposing the data sharing movement. In addition, there was a perceived sense of legal uncertainty from legislations governing the sharing of health data, which adds unreasonable burdens on individual researchers, who are often unequipped to deal with such facets of their data sharing activities.

## Introduction

Health research is gradually breaking off from a long tradition of relatively closed and individualistic scientific endeavors to a new movement of data sharing, open science and collaborative partnerships (1–3). This is facilitated by the fact that the healthcare domain has become a data rich environment, with the increasing availability and complexity of data sources—gradually framed under the terms *big data* or *big biomedical data*, and the need to push the healthcare agenda toward precision medicine (4–6). Given the complexity of these data sources, conventional scientific approaches fall short in providing in-depth insights on the causality of diseases and subsequently the ability to adequately tailor healthcare interventions in this data deluge (7). Indeed, in contrast to industrial settings where *big data* approaches are well-implemented due to inherent characteristics of datasets used (e.g., acquired secondarily, high volume, low information density and easily accessible), healthcare datasets have a different status (6).

Healthcare data are more complex, expensive, predominantly kept in silos with high information density, and under additional legal protection due to their sensitive nature, which altogether render them less accessible to benefit from big data methods (6). Nevertheless, the healthcare data for the entire world in 2020 is alleged to be around 2,314 exabytes, representing a fifteenfold increase from 2013^1^. Therefore, present-day health research is also transitioning to the fourth research paradigm, termed “data-intensive science,” where new technologies and techniques are required to make sense out of this data deluge (7–9). In this new paradigm, the expected benefits in terms of improved research and healthcare outcomes can be achieved if data are shared easily in an interoperable manner, from a multitude of stakeholders, for collaborative research, diagnosis and treatment (7, 10). This implies that the sharing of data is one of the *foundations* of the new paradigm. However, the data sharing status in healthcare has been relatively weak (1) in comparison to other domains [e.g., genomics or astronomy (11, 12)]. For instance, one of the current data-intensive science initiative is the *Global Alliance for Genomics and Health* (GA4GH) (13), which was founded in 2013 and aims to promote the responsible sharing of genomic data around the world, whilst abiding to a human rights framework^2^.

The presumed benefits of sharing data for clinical care and research have been extensively discussed in the scientific literature. Some of them include: (i) an increase in scientific discoveries [e.g., testing of new hypotheses (14)] and their subsequent uptake in routine clinical practice, (ii) providing better care for patients, (iii) a reduction in research waste, and (iv) the verification and reproducibility of research findings to ensure research integrity and transparency (1, 15). In addition, one of the important tangible goals of data sharing is to provide insights on rare diseases, where data are often limited. For instance, the *Matchmaker Exchange* (16) is a data sharing platform that allows the discovery of genes related to rare diseases through matching algorithms. In this regard, it adopts a federated approach in which different and autonomous databases (e.g., genotype and phenotype) are connected through a standard application programming interface (16). This platform has already helped to diagnose rare diseases (17).

However, data sharing also generates challenges for both data subjects (18) and primary data collectors. For instance, the *International Committee of Medical Journal Editors* (ICMJE) considered data sharing as an ethical imperative for the risks taken by participants enrolling in clinical trials, and it has taken over the years a strong stance on normalizing the sharing of clinical trials data (14, 19). The ICMJE also noted the persistence of some unsettled issues such as safeguarding researchers' interests in terms of proper attribution mechanisms for sharing data. Such issues can negatively influence data sharing requirements often present in academic publishing or in registers for clinical trials (20, 21). In this perspective, the ICMJE also recommends that, whenever possible, original data collectors should be offered the opportunity to collaborate on secondary research projects if their data are being used or at the minimum, their data collection efforts have to be acknowledged^3^.

In spite of increasing efforts aimed at promoting data sharing in academic and research enterprises, resulting conflicts are sometimes portrayed simplistically as a black-and-white issue, where self-interests of primary data collectors are pitted against the idea of reusing data for the public good (22). To help solve this impasse, technical aspects to ease the sharing of data have received much attention, but there has been a lack of studies capturing our understanding of the incentives or disincentives that influence stakeholders' behavior with respect to data sharing (15, 23), especially from a systemic perspective. Such limited insights can hamper the efforts to promote data sharing by neglecting some cultural peculiarities, practices and interests of these stakeholders (10, 15, 24). Furthermore, the individual behavior of stakeholders with regard to data sharing is influenced largely by systemic factors (e.g., institutional policies or practices etc.) (25).

Although incentives for researchers to share data have not been investigated in depth (1), some factors discouraging data sharing from the researchers' own perspectives have been identified. For instance, preparing and managing datasets for secondary use prove to be a time-consuming and costly process (10, 24, 26). Original data collectors often do not possess the required knowledge to successfully carry out these tasks (27). Moreover, the competitive environment of research does not foster a data sharing culture, since advancement in the academic career is linked primarily to the number of peer-reviewed publications in high impact factor journals, rather than on the number of research datasets made available for reuse (28). Besides, there is a lack of trust in the system and a real fear of getting scooped by external researchers gaining access to the data or that the data will be misused or misinterpreted by data recipients (10, 24, 26). The current systemic incentives—such as data sharing mandates from funders, journals or governments—cannot one-sidedly solve this complex issue (29). In this regard, Whitworth advocates for a broader collaborative approach where all involved stakeholders (e.g., researchers, ethics committees, journal editors and governments) can voice their opinions and reach consensus on instilling a data sharing culture (29). This is particularly important given that unilateral data sharing mandates do not address one central ethical principle, which is *fairness* for the primary data collectors (28).

*Fairness* is simply defined as, “the quality of treating people equally or in a way that is right or reasonable”^4^. In 2015, *fairness* was one of the guiding values put forward by a Committee of the *US Institute of Medicine*, to ensure that clinical trial data are shared in a responsible manner (30). It was highlighted that all involved stakeholders (from trial participants to researchers and sponsors) have an interest to ensure that *fairness* guides data sharing activities (30). Nevertheless, perceptions of fairness from involved stakeholders might differ based on their respective interests. On the one hand, trial participants might be more concerned in ensuring that no societal groups are unfairly discriminated in reaping the health benefits brought by these clinical trials (30). On the other hand, researchers might find it not only more important and fair to protect their interests relative to the data that they collected (e.g., if secondary publications are to be expected), but also to receive safeguards and due credit for the invested efforts, time and intellectual resources once their datasets are made available for reuse (30). Indeed, from the perspectives of primary data collectors, “any unsolicited and unsanctioned use of their data shall be seen as unfair” (31). Therefore, it is important that burdens and benefits of data sharing are fairly distributed between the original data collectors, the data recipients and ultimately, the data subjects. If the *fairness* dimension toward the primary data collectors is not addressed, these might only provide datasets that fulfill the minimum quality standards required for sharing, which can be at the detriment of high quality secondary research (28).

At the same time, it is also important to consider the ethical imperative of data sharing, which “requires that data that can be used for research purposes and research results should be made available for further research use to advance the common good of scientific knowledge” (32). Therefore, timely access to health data for secondary research purposes should not be discriminatory, delayed or restricted without due justification (32). In this aspect, *fair data sharing* should not be confused with the FAIR Data principles (33). Indeed, the latter consists of four principles (i.e., Findability, Accessibility, Interoperability and Reusability) that need to be applied to not only scholarly data but also to other tools used in the generation of such research data (e.g., algorithms and workflows) for subsequent re-use in either human-driven or machine-driven initiatives (33).

In this paper, we tackle the topic of *fairness* with respect to data sharing practices by presenting the relevant findings of a qualitative study we conducted with Swiss stakeholders. We focus in particular on matters of systemic elements that impact on fairness in data sharing. These are those elements that are inherent to the overall healthcare system, including the research domain and regulatory frameworks, that affect the fair sharing of health data, rather than those elements being mainly connected to individual motivations of the original data collectors.

## Materials and Methods

### Ethics Approval and Consent to Participate

The present study is part of a larger project titled “advancing SMart solutions and eliminating barriers for the Acquisition, Analysis, and Sharing of Health data in Switzerland” (SMAASH). The project falls outside the scope of the *Human Research Act* (HRA)^5^—the Swiss law on medical-related research—and thus does not require ethical approval according to Swiss regulation. This was confirmed by the cantonal ethics committee in Northwest and Central Switzerland, to which the project was nevertheless submitted (reference number: EKNZ req-2017-00810). The committee commented that the project does not pose any health risks to participants and it satisfies both the general ethical and scientific requirements. Study participants were then recruited via email for semi-structured interviews and informed about the nature and objectives of the study, the expected duration of the interview, and measures that would be taken to ensure confidentiality. Participants orally agreed for the interviews to be audio-recorded so that transcripts, with no personally identifiable information, could be created for further analysis. Upon request, some participants reviewed their transcripts for accuracy prior to the start of the analysis.

### Research Team and Reflexivity

The research team consisted of two PhD candidates in biomedical ethics (LDG and AM), a senior researcher (TW) and the two principal study investigators (TP and BSE). After receiving training in qualitative research and acquiring the necessary interviewing skills, LDG and AM conducted the semi-structured interviews. LDG has a background in medicine and global health, while AM in law. They were supported during the analysis of the interview transcripts by TW and BSE. TW and BSE are both established scholars in qualitative health research. TP is an expert in the assessment of health services and quality of care. Constant supervision by TW, TP, and BSE helped limit possible bias in the interpretation of the data. Since study participants were mostly experts in their respective fields and often did not have a “neutral” view on the topic, it was important for the two interviewers to adapt their epistemological position throughout the interview (from co-expert to lay person or critic and vice-versa) (34). This served to expose and challenge assumptions made between the interviewer and the interviewee(s).

### Recruitment and Characteristics of Participants

Purposive and snowball sampling techniques were used to recruit participants for the semi-structured interviews. The eligibility criteria for participation are researchers and policymakers working in the Swiss healthcare domain or individuals, with a relatively high position, involved in the collection, curation, sharing, linkage, and management of health datasets (e.g., registries, hospital IT infrastructure, national/regional data initiatives or hospital directors). As part of the overall aims of the SMAASH project, we carried out a systematic review (35) on projects collecting, sharing, and linking health data. Through projects analyzed in the review, we were able to recruit some of our participants. The response rate was 83%. We conducted 43 semi-structured interviews with 48 participants, since four of the interviews were one-to-two (*n* = 3) and one-to-three (*n* = 1). Our study participants had rich and diverse backgrounds (see Table 1). An interview date was scheduled with the consenting participants and further information and explanation were provided prior to the start of the interview.

**Table 1:** Characteristics of study participants.

**Type of Participants (n/%)**	**Language Speaking Regions in Switzerland**		
	German-speaking^****^ **n (%)**	**French-speaking** **n (%)**	**Italian-speaking** **n (%)**
Researchers^*^ (28/58.3%)	17 (60.7%)	8 (28.6%)	3 (10.7%)
Person involved in politics^**^ (10/20.8%)	9 (90.0%)	1 (10.0%)	0 (0.0%)
Person with a high position in either a health register/IT infrastructure/national initiative on health data or as hospital director^***^ (10/20.8%)	8 (80.0%)	1 (10.0%)	1 (10.0%)

* *abbreviated as “R” in results section*.

** *abbreviated as “P” in results section*.

*** *abbreviated as “H” in results section*.

**** *some cantons (i.e., states) are bilingual but are classified in Table 1 as monolingual based on the main language spoken (e.g., Bern as German-speaking)*.

### Data Collection

LDG, AM, TW and BSE developed the interview guide (Appendix 1), which was then pilot tested to ensure that each question was easily accessible and understandable to a broad audience and modifications were made accordingly based on feedbacks received. As of May 2018 to September 2019, LDG and AM independently conducted the 43 semi-structured interviews. The duration per interview ranged from 38 to 131 min. The majority of the interviews were conducted in English (*n* = 37), while the remaining few were conducted either in Italian (*n* = 2), German (*n* = 3) or French (*n* = 1), based on the preferences of the interviewees. All audio recordings were treated confidentially, verbatim transcribed, but omitting details in the transcripts that could help identify the interviewee.

### Data Analysis

We conducted a thematic analysis, guided by the six-step framework devised by Braun and Clarke (36). The recordings were transcribed and analyzed using the qualitative analysis software, MAXQDA (versions 18 & 20). Three members of the research team (LDG, AM, and TW) conducted a series of preliminary meetings to discuss the transcripts of the first seven interviews. The result of these meetings led to the development of a coding tree and the identification of themes/subthemes pertinent to the overall aim of the SMAASH project, which is to identify barriers and facilitators with respect to the processing of health data (e.g., collection, sharing, and linkage activities). The coding tree was further developed and finalized following the subsequent analysis of the remaining interview transcripts. LDG and AM coded individually the remaining transcripts and concertedly discussed with TW the identification of new themes/subthemes during a last series of meetings aimed at analyzing a sample of 15 additional transcripts. When the data corpus was coded with the finalized coding tree, another meeting was held with all the authors to discuss the macro topics within the data.

In this manuscript, only the themes/subthemes related to the macro topic of systemic fairness in data sharing are considered. LDG created a dataset where data extracts pertinent or relevant to the perception of systemic fairness guiding data sharing were gathered. This dataset was then reanalyzed from a “theoretical” or deductive perspective (36). That is, LDG re-analyzed the data extracts into themes that address the topic of systemic fairness and identified two themes and two subthemes. The quotes used in the results section were edited grammatically and non-English data extracts were translated. In a subsequent meeting, the authors discussed, refined and agreed on this final set of identified themes and subthemes for this study.

## Results

### Matters of Systemic Fairness

**Figure 1:**
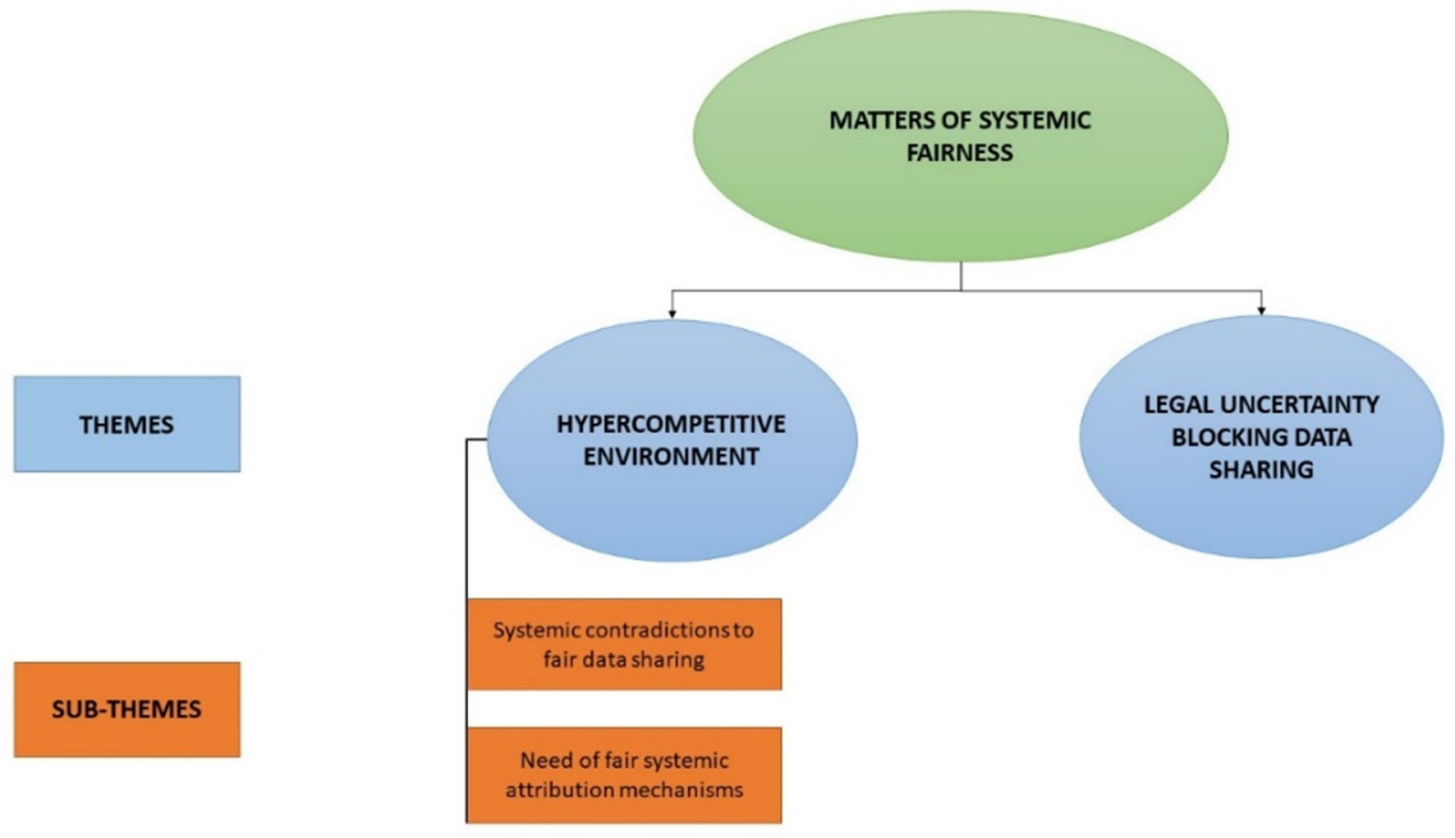
Themes and sub-themes on perceived systemic constraints for the fair sharing of health data.

Two themes relevant to systemic fairness in the sharing of health data emerged in our study. These are (i) *hypercompetitive environment* and (ii) *legal uncertainty blocking data sharing* (Figure 1).

### Hypercompetitive Environment

#### Systemic Contradictions to Fair Data Sharing

*Systemic contradictions to fair data sharing* comprise those barriers posed by current practices and policies, which (i) foster competition in the research context for limited resources, and (ii) hinder the fair sharing of health data. For instance, one researcher highlighted that unwillingness to share data is not simply a matter of selfishness and ego, but a failure of the system to cater for researchers' needs. She further explained that academic survival is a “real fear” that researchers have to face during their career, and it would be incorrect to attribute their protective data sharing behavior solely to their ego and self-centeredness.

≪*I think there is a real fear, I think it is fear that they [researchers] are going to lose their spot, they are going to lose their place you know […] This is our system, I think it brings all kinds of… you know…impure intentions ultimately…you can't blame individuals. It's the system that we are working in and the system itself is a bit sick*… ≫ – ***43REngPA, R***

Other participants also linked the reluctance to share data with the fact that wrong incentives for career advancement are provided, which ultimately lead researchers to adopt individualistic behaviors. One participant had a relatively pessimistic view about the academic system and underlined the tremendous difficulties associated with trying to change the way it currently operates, unless there is the political will to do it.

≪…*the credo in research is still “publish or perish” […] you need to publish papers to get additional new funds. And this system I think will not change unfortunately in the next decades. It is like…it is very difficult to change. So, if we want to change something I think you should start from the political sides or from the top one*. ≫ – ***2HEngPA/B, H***

The way the academic system incentivizes career advancement was perceived by some participants as being contrary to the movement of open science and data sharing. Participants stated that academic excellence is being assessed on certain quantitative performance metrics, driven primarily by publication pressure, which contrasts not only with the idea of data sharing (since researchers often end up being in competition with other researchers for publications based on their own data), but it also exposes the researcher to unfair practices (e.g., run the risk of getting *scooped*).

≪ *The only concern we have is that the current academic system wants us to excel in our field attested by publications made in peer-reviewed journals, the issue is that we do not want to have projects that are then, on the pretext of sharing, which are then stolen by others*≫ – ***13RFrePA, R***

≪ …*publish or perish is one reason, but also maybe I have some ideas, I want to test with the data first and I don't want to give others the opportunity to take away my ideas just because I didn't have the time to do it right now. So… I completely see it that you don't want to share your data the day you get them*≫ – ***22REngPA, R***

Therefore, from the perspectives of some interviewees, the academic system forces researchers to take a counterintuitive inclination with regard to the timely sharing of data. For example, researchers might be unwilling to share datasets right after publication if they have not been able to fully exploit them to answer interesting research questions, in particular given the time and efforts put into collecting and curating the data. This is particularly true if there are risks that these questions could be answered quicker by external researchers, thereby depriving the original data collectors from a high quality publication:

≪*I mean of course we are all a bit reluctant [to share data] because we perhaps or most people perhaps share a certain fear that you know that others might use data for things that we could do ourselves and then kind of use our data to generate analysis we would like to generate at a later point in time and […]in the end it would be their publications and not ours*≫ – ***41REngPA, R***

≪…*for many studies, you've spent four years for collecting data, and then before you would have time to analyze and publish it/and at that stage, people want the datasets because they don't spend any time on collection, they publish even the results quicker than you do. That's a reason why many researchers will be reluctant to share datasets*≫ – ***16REngPA, R***

≪*…well first when you collect the data, you have to make a protocol to make it funded, to go through the ethics committee. So it's very big work and it needs resources, financial and human resources. So in the end, you know you've done all the work, you generated a beautiful database with everything cleaned, and you just…you know that someone asking you: “well give me your database and we will do ten papers on your work and you will not be in the authors. You will just be maybe in the acknowledgments and that's it”* ≫ – ***17REngPA, R***

≪*Because you might be working on something still especially with big studies, that sometimes takes a long time until they find the time to analyze the data that they have. And they put a lot of effort in collecting these data and then their hands are often bound to exploit them in a timely fashion. So I think for them that it's difficult if then other people come and just, you know, just exploit*≫ – ***20REngPA, R***

Moreover, if quantity of publications—and not only their quality—is an important element to help advance one's own career, data sharing can then be perceived as a liability. For instance, one researcher highlighted the practice of hiring researchers with more publications.

≪…*The ways we incentivize people are quite wrong. They are really pushed toward individualism and you know, you have to have the best curriculum with thousands of papers, even if you never participate really in the papers […] They will hire a person publishing more than a person publishing good, good work. Even here…*≫ – ***17REngPA, R***

#### Need of Fair Systemic Attribution Mechanisms

Some participants noted that there are no fair systemic attribution mechanisms for researchers and other people involved in the management, curation and preparation of datasets for secondary use. For instance, one participant underlined the difficulty in ascertaining the reasonable and acceptable way of giving credit back to the original data collectors for their work in providing the datasets.

≪*Well the question is what kind of credit, right? Because if I get data from somebody and I use it, I do have to say/ I mean I give credit by saying: “I use this data from group XYZ”, so in that way I already acknowledged that somebody else put the work in it and I just do the analysis. But that might not be enough credit for a researcher*≫ – ***20REngPA, R***

Others highlighted the need for a systemic change regarding reward mechanisms to ensure that all people involved in data sharing activities receive the rightful acknowledgment for their work. One participant reported having offered co-authorship to data managers as a compensation for their contribution in ensuring the good quality of datasets for sharing purposes.

≪*Our academic system is now built on impact factors in papers and there are some other ideas of valuing some other work as well. I mean preparing a dataset is an excellent example of those people [who] never get credit. And we've been working on this [name of cohort] for years. We asked people to add to the author list the [name of cohort] study group. So we get as a reference that this is [name of cohort] data. I think we should think about a system which gives credit to people who actually collect data, manage data and [en]sure the quality…*≫ – ***23REngPA, R***

It was also noted that there is a need for funding agencies to develop and implement attribution mechanisms that consider not only the publication record of researchers but also their data sharing activities as an additional evaluation criterion for grant approvals. Indeed, even if funders provide some sort of financial compensation for data sharing activities as part of the overall project budget, acknowledging data sharing activities of researchers is perceived as a fair incentive, in particular in terms of opportunities for career advancement.

≪…*it [data sharing] should be acknowledged by those who have the power, a special science foundation, funding agencies, that you sort of get some benefits or some credits*≫ – ***25REngPA, R***

≪*I think the [Swiss funding agency] now says: “You need to have a data plan and you need/ you can put it into the budget to produce that.” The problem is that for researchers that sort of effort is not moving your career very quickly forward. So this is also/ at the end of the day we then need down the road some matrix that shows: This is a researcher that is nicely sharing data and this is a researcher that doesn't nicely share data. That sort of incentives then also need to come sooner or later. It's part of your well-standing of a researcher that you have an established record of making data available if someone asks*. ≫ – ***31REngTA, R***

One researcher highlighted that although certain attribution mechanisms exist for crediting researchers who made their datasets available for re-use, these are not widely implemented in the academic system. In addition, the researcher underlined that if data receivers promise to credit the data collection efforts of the original data collectors by citing the unique persistent identifiers of datasets, it could be an incentive for the original data collectors to grant access to the data.

≪*It [dataset] needs an ID like a paper as well. It already exists but not a lot of people use it. Each dataset needs an ID plus each dataset needs meta-data which describe the data and there should be the name of the author or the research team or the institute or whoever […] If it's possible you give access to the data to new research teams given that they reference you as the source of the data*. ≫ – ***23REngPA, R***

### Legal Uncertainty Blocking Data Sharing

Legal uncertainty resulting from the complex and fragmented legal landscape can be at odds with data sharing, since it can delay or even prevent access to datasets that would have otherwise been accessible for secondary use.

Some participants expressed frustration on how multi-cantonal or nationwide research projects and registries were particularly vulnerable to not only different interpretations of the same piece of legislation, but also to differing data protection requirements imposed by cantonal laws. The allegedly unclear legal situation resulted in a fragmented data sharing climate, where researchers' motivations for sharing health data were perceived as being obstructed by uncertainty resulting not only in ascertaining which data protection requirement should prevail over the others (e.g., federal or cantonal), but also by the myriad of cantonal data protection regulations. For instance, one researcher found it difficult to know under which circumstances cantonal or federal law should prevail, highlighting the complexity to navigate between these different legislations. When referring to multi-cantonal projects, the same researcher expressed difficulties in ascertaining under what conditions a specific cantonal law would apply.

≪*Which legal bases apply in which context and what is the overlap? There is also a national law, there are cantonal laws, and so what law applies to which situation? … I'm not a lawyer, am I?* ≫ – ***35RGerPA, R***

≪*So there are at least three [participating] cantons. Then there is always still the question of which cantonal law is applicable, right? Is [it] the one of the research site? Is [it] the one where we collect the data? […] So yes, we now do it the way we do it, but if a lawyer looked at it properly, she might have questions about it, right? I do not know* ≫ – ***35RGerPA, R***

As a consequence of the fragmented legal landscape, another researcher, with experience in managing a health database, expressed annoyance regarding the disparities in evaluation of the same research project by different cantonal data protection officers.

≪*Yes first to the situation in Switzerland: I realized that some… It's not everything just black and white. And I realized that some [cantonal data protection officers] are very liberal and some others are not at all. I realized that there are some differences how they actually look at a specific research project or registry. So sometimes it's rather difficult to know what we are allowed to do…*≫ – ***23REngPA, R***

Such uncertainties had also an impact on the scope of research projects. For instance, one researcher with some experience in managing several disease registries, was frustrated when she tried to navigate through the complex fragmented legal and regulatory landscape for her different projects.

≪…*this unclear legal situation with many questions […] one is the ethics committee do this way, and the other this way for the same thing […] the lawyers from the Federal Office of Statistics have different interpretation of the same laws as the lawyers from the Federal Office of Health, and the lawyers from the ethics committees and of the hospitals… this is complicated and usually then you can choose to do what all the lawyers agreed, which is the minimal*≫ – ***16REngPA, R***

Some participants also experienced difficulties determining whether their data sharing activities were legally compliant. One participant talked about the legal uncertainty arising from collaborations within European research projects, in particular since the application of the European *General Data Protection Regulation* (GDPR) in 2018. It was difficult for the participant to find out whether data processing activities within Switzerland abide to GDPR standards, which would have allowed the sharing of health data with a European partner. Faced with such legal uncertainty, he resorted to not sharing any personally identifying information with European partners.

≪…*I'm looking forward that Switzerland actually gives me some guidelines how to do it with international data. I need to know what am I allowed to do with European data on servers in Switzerland and the other way around. What exactly am I allowed to send to a server in the EU with clinical data from Switzerland or patient data. The easiest way is to keep all identifying information in Switzerland*. ≫ – ***23REngPA, R***

Another researcher has had trouble determining whether the tools used in sharing datasets with internal or external partners were legally compliant (e.g., sharing datasets via email).

≪…*Of course, we can always ask ethics committees if it's allowed but the tools we are using to share data, and this can be something like sending an email with a datasheet. It can be…you know there can be misuse of it. And nobody knows how secure it is*≫ – ***19REngSA, R***

It was also interesting to note that one person responsible for hospital data management had legal concerns regarding data security measures taken by the data recipients. For instance, she highlighted that collaborations between University Hospitals and Universities created a climate of legal uncertainty due to the differing data security measures adopted by these two stakeholders.

≪…*I mean the hospital has its extremely strong firewalls and as the (Swiss University Hospital) is a hospital and works closely together with University but it's not the same […] we do not really know how it's law confirmed that we can own and hand over data*. ≫ – ***14HEngPA, H***

## Discussion

This qualitative study provides important policy-making insights on the perceived systemic determinants of fairness in the health data sharing ecosystem from the perspectives of Swiss stakeholders (mostly researchers). Our stakeholders were mostly in their mid- to late-career stages. These determinants include systemic constraints to the sharing of health data induced by hypercompetition in the Swiss research domain (i.e., systemic contradictions and the lack of fair attribution mechanisms for data sharing), and the perceived uncertainty arising from the legal and regulatory framework governing data sharing activities therein. The latter resulted in different interpretations of the legal and regulatory frameworks, and thus the need for stakeholders to sometimes err on the side of caution and give up on certain aspects of their respective projects or some collaborative research activities.

The hypercompetition between researchers for limited resources and opportunities (e.g., obtaining grants from funding agencies, academic recognition and career advancement) has been extensively discussed in the scientific literature (10, 25, 37–39). Hypercompetition is generally portrayed as having both positive and negative influences on research (40). However, our participants mostly considered the negative influences of hypercompetition on the sharing of health data, underlining increasing systemic tensions between academic survival [e.g., from “publish or perish” to “funding or famine” (37)] and the movement of open science and data sharing that is being introduced also in the Swiss context^6^.

In a 2019 report to the Swiss National Science Foundation (SNSF) on Open Research Data, a survey revealed that about 75 percent of Swiss researchers actually share their data (e.g., through personal request and openly on journal, webpages and repositories). Some of the reasons given for not sharing data were (i) the need to publish before data sharing is considered, (ii) lack of time to carry out data sharing activities, (iii) fear of getting their research scooped and (iv) not receiving due credit for the shared datasets (41). The need to publish before making data available for reuse was also one of the top barriers identified by Tenopir et al. (10) among scientists globally. These reasons are in line with findings of our qualitative study and could be explained by how the academic system primarily assesses academic performance of researchers via their publication records and their ability to secure grants from funders, rather than considering their data sharing activities. In this regard, Switzerland is not an exceptional case and mirrors the academic evaluation processes of other countries (12, 38).

To reduce the negative impact of hypercompetition on data sharing, some of our participants proposed that there is a need to provide systemic attribution mechanisms as an incentive for researchers and data managers involved in data management and sharing activities. Additionally, such a measure would allow researchers and institutions to not only receive due credit for their contribution to data sharing activities but it would also be another yardstick that could provide a better evaluation of their academic performance. Indeed, although present quantitative performance metrics (e.g., number of peer-reviewed publications) were initially used as a fair basis to distribute the scarce or finite resources (e.g., grants or academic positions) to deserving researchers, they have reportedly degraded the proper functioning of the academic reward mechanism (37).

For instance, the quantity of publications, under the so-called “publication pressure,” is sometimes prioritized for distribution of finite funding resources. This was also pointed out by some of our experts, highlighting its contribution to the adoption of self-centered behaviors by researchers, which subsequently hinder the timely sharing and reuse of research data. Such phenomenon can be partly explained by a reformulation of *Goodhart's* Law by Marilyn Strathern, who stated that “when a measure becomes a target, it ceases to be a good measure” (37, 42). Therefore, even if data sharing activities are to be added as an integral quantitative performance metric in Swiss academic performance evaluations and others, it is paramount to ensure that researchers and other stakeholders do not end up producing a high number of low-quality datasets just to increase their chances in securing grants or academic positions in the hypercompetitive Swiss environment.

One way data sharing activities can be encouraged without risking to turn them in an over-individualistic metric was proposed by Pierce and colleagues (12) by highlighting that it is crucial to explicitly acknowledge the scientific value of shared datasets as an additional recognition for data generators irrespective of their publications. In this regard, they proposed to attribute and link unique persistent identifiers (UPI) to both the data generators (e.g., ORCID for scientists) and the shared datasets (e.g., digital object identifiers). Citation metadata, for the original and any subsequent publication using the shared dataset (by either the data generators or external researchers), would need to include the dataset's UPI, and the citations added to CrossRef. These would give credit to the original data collectors every time their shared dataset is being used (12). As dataset citations are neither fully adopted nor do they capture the full complexity associated with data usage, there is also the need to develop standardized data usage metrics (e.g., the number of times a dataset has been viewed or downloaded) to better capture and measure the impact of shared datasets in moving forward research (43). If such data usage and data citation/reuse metrics are widely implemented within the research arena, they will not only offer a fairer evaluation of the academic performance of researchers (44), but they will also encourage the sharing of high quality datasets. Indeed, competition in academia is likely to be inevitable in the foreseeable future, given that resources are finite. However, it becomes a liability if it turns into a race to the bottom. By steering academic competition in the right direction through adequate and fair incentives, it could give a new impetus to data sharing activities.

With regard to the Swiss regulatory framework, uncertainty in terms of legal disagreement and legal compliance were perceived by our participants, which influenced the fair sharing of health data. One of the contributing factors is that data protection regulations are elaborated broadly to cover many situations but they lack specificity when applied to a particular context (45). Furthermore, even if there are research exemptions or research-specific rules that are implemented within certain pieces of legislations (e.g., the GDPR at the European level) or the Swiss Federal Act on Data Protection (FADP), it is still unclear for researchers how to implement these exceptions in practice (46, 47). These issues were reflected in our study where participants experienced difficulties in ascertaining whether their data sharing activities were operating within legally acceptable margins. Another contributing factor to the perceived legal uncertainty is the multitude of data protection regulations present in the Swiss legal landscape and the difficulty in identifying which specific regulation would supersede the other in a particular context. In the current Swiss legislative framework, solving this legal uncertainty requires a case-by-case approach (46), but this uncertainly slows down data sharing.

As mentioned above, fair data sharing has been proposed as an essential guidance for the exchange of health data, that data be usable to produce important research findings or promote the improvement of healthcare are promptly shared (32). If the regulatory landscape does not necessarily provide an additional layer of data protection, but rather has the main result of hampering data exchange, corrective measures should be taken. This does not necessarily require changes in legislation: it could be achieved, for example, if host institutions offered researchers the necessary training, infrastructure and legally-compliant data transfer mechanisms to ensure that they can easily determine how to operate within legally acceptable margins. One way this could be achieved uniformly across national and international institutions is through the implementation of codes of conduct or through an adequacy model, such as the adoption of data protection certification mechanisms specifically designed to facilitate the sharing and reuse of health data for research purposes, while reducing privacy and informational harm risks for data subjects (45). In this regard, policy-makers in the healthcare and research fields could learn from experiences of successfully implemented data protection certification mechanisms in other domains.

For instance, the *Asian-Pacific Economic Cooperation*'s Cross-Border Privacy Rules System (APEC CBPR) is an established data protection certification system for trade, guaranteeing the legal compliance of companies with regard to data protection while ensuring collaboration with local governments. Indeed, “certified companies and governments are working together to ensure that when personal information moves across borders, it is protected in accordance with the standards prescribed by the system's program requirements and is enforceable across participating jurisdictions”^7^. Such an approach allows a leveling-up of data protection requirements across participating organizations, while ensuring that the local legislation within which these companies are operating is respected (48, 49). The GDPR also proposes the implementation of data protection certifications under a voluntary basis (see Art. 42)^8^. Therefore, a specific data protection certification mechanism for health research could also be useful to facilitate data sharing between institutions (45) while contributing to more fair conditions and less frustrations in the data sharing process. Moreover, this approach may also reduce the risk of imposing unfair additional financial and time constraints on already resources-limited researchers and healthcare professionals in ensuring legal compliance of their data sharing activities.

### Limitations

This qualitative study has several limitations. Firstly, the low reporting of benefits of hypercompetition from our interviewees may result from the way interview questions were formulated, being more prone to discuss the barriers to health data sharing. Secondly, most of our interviewees were mid-career to late-career participants, which could have not prioritized some systemic constraints to the fair sharing of health data more pertinent to early-career researchers or healthcare professionals. Thirdly, matters of individual motivations to the fair sharing of health data have not been tackled in this article because of the richness of the data and had to be analyzed separately as part of another publication within the framework of our research project. In addition to these topic specific limitations, we do not claim our work to be generalizable to other contexts and that social desirability bias may also have played a role in the information that we received. Moreover, some of our interview sessions had to be adapted to the needs and limited availability of our interviewees, which led to some sessions being one-to-two or one-to-three interviews.

## Conclusions

The open science and data sharing movements have their *raison-d'être* in improving and facilitating health research, but it will be difficult to fulfill such aim unless careful consideration is given to unfair systemic inconsistencies undermining such initiatives. This qualitative study has brought into light two main systemic barriers that can undermine the fair sharing of health data from the perspectives of Swiss stakeholders. First, hypercompetition in the Swiss academic system has perverted the way finite resources are distributed to stakeholders which led them to adopt individualistic behaviors and refrain from sharing datasets. Second, a perceived legal uncertainty in the complex Swiss regulatory landscape has limited the sharing of health data by imposing unfair conditions on researchers, leaving it up to individual researchers to deal with specific interpretative and implementation aspects of the different pieces of legislation, a competence that many do not possess. As long as stakeholders believe that their legitimate interests in their datasets are not fairly safeguarded by the system, data sharing will remain difficult and the objectives of open science will be hard to achieve.

## Data Availability Statement

The de-identified dataset used to support the conclusions of this article is available upon reasonable request to the corresponding author. To prevent the re-identification of our study participants, full transcripts cannot be shared since confidentiality was guaranteed as a prerequisite for participation.

## Ethics Statement

Ethical review and approval was not required for the study on human participants in accordance with the local legislation and institutional requirements. Written informed consent for participation was not required for this study in accordance with the national legislation and the institutional requirements.

## Author Contributions

LG and AM were involved in the data collection part of the study. All authors were involved in the analysis and in defining the themes pertinent to systemic fairness in the sharing of health data. LG wrote the first draft of the manuscript, which was then reviewed and edited by the authors. The manuscript was then finalized and approved for submission by all authors.

## Conflict of Interest

The authors declare that the research was conducted in the absence of any commercial or financial relationships that could be construed as a potential conflict of interest.

## Footnotes

^1^EMC Digital Universe and Analysis by IDC (2014): “The Digital Universe of Opportunities: Rich Data and the Increasing Value of the Internet of Things,” as cited in *Stanford Medicine 2017 Health Trends Report: Harnessing the Power of Data in Healthcare* by Stanford Medicine (p.4). URL: https://med.stanford.edu/content/dam/sm/sm-news/documents/StanfordMedicineHealthTrendsWhitePaper2017.pdf. [Accessed date: February 17, 2021].

^2^Global Alliance for Genomics and Health (2021): “Enabling responsible genomic data sharing for the benefit of human health.” URL: https://www.ga4gh.org/. [Accessed date: January 21, 2021].

^3^ICMJE Editors (2021): “Clinical Trials.” URL: http://www.icmje.org/recommendations/browse/publishing-and-editorial-issues/clinical-trial-registration.html. [Accessed date: January 22, 2021].

^4^Cambridge English Dictionary (2020): “Fairness.” URL: https://dictionary.cambridge.org/dictionary/english/fairness. [Accessed date: June 4, 2020].

^5^The Federal Council (2020): “Federal Act on Research involving Human Beings.” URL: https://www.admin.ch/opc/en/classified-compilation/20061313/index.html. [Accessed date: June 5, 2020].

^6^Swiss National Science Foundation (2020): “Open Science”. URL: http://www.snf.ch/en/researchinFocus/dossiers/open-science/Pages/default.aspx. [Accessed date: November 5, 2020].

^7^Asia-Pacific Economic Cooperation (2019): “What is the Cross-Border Privacy Rules System?”. URL: https://www.apec.org/About-Us/About-APEC/Fact-Sheets/What-is-the-Cross-Border-Privacy-Rules-System. [Accessed date: November 5, 2020].

^8^Intersoft Consulting (n.d.): “Art. 42 GDPR Certification”. URL: https://gdpr-info.eu/art-42-gdpr/ [Accessed date: November 5, 2020].

## Supplementary Material

Appendix 1 Interview guide for Swiss stakeholders.

## Abbreviations

APEC CBPR: Asia-pacific economic cooperation's cross border privacy rules system
EHR: electronic health record
FADP: federal act on data protection
GDPR: general data protection regulation
HRA: human research act
ICMJE: International Committee of Medical Journal Editors
SNSF: Swiss National Science Foundation.
